# Contrast-enhanced computed tomography for optimizing the outcomes of pulmonary vein isolation with cryoablation -the role of isolation of PVs including carina-

**DOI:** 10.1007/s10840-021-01052-5

**Published:** 2021-08-25

**Authors:** Yusuke Sakamoto, Hiroyuki Osanai, Yuki Tanaka, Shotaro Hiramatsu, Hikari Matsumoto, Kensuke Tagahara, Hirotaka Hosono, Shun Miyamoto, Shun Kondo, Takahiro Kanbara, Yoshihito Nakashima, Hiroshi Asano, Masayoshi Ajioka

**Affiliations:** Department of Cardiology, Tosei General Hospital, 160 Nishi-Oiwake-cho, Seto-city, Aichi 489-8642 Japan

**Keywords:** Atrial fibrillation, Cryoablation, PV isolation, Contrast-enhanced CT, Isoproterenol

## Abstract

**Purpose:**

Compared with conventional pulmonary vein isolation (PVI) with radiofrequency ablation, PVI with cryoballoon is an easier and shorter procedure without reconnection, particularly in the superior pulmonary vein. However, the durability of the cryoballoon may be reduced due to anatomical factors and the position of the pulmonary vein (PV). Further, inadequate isolation of the carina leads to recurrence of atrial fibrillation (AF). We aimed to determine whether using contrast-enhanced computed tomography (CT) for patient selection improves the early success rate and prevents the recurrence of AF in PVI with cryoballoon.

**Methods:**

We evaluated patients who underwent ablation for paroxysmal atrial fibrillation in our hospital between July 2019 and November 2020. After excluding patients with contraindications for cryoablation, 50 patients were selected through visual inspection of the results of preoperative contrast-enhanced CT. A treatment plan was established, and the clinical course and outcomes were followed up.

**Results:**

Of the 200 PVs of the 50 patients, only 8 PVs (4%) were incompletely isolated with a single cryoablation. Six of the eight PVs were successfully isolated with additional cryoablation. Only 2 patients (4%) underwent additional PVI with radiofrequency ablation. Four patients had AF recurrence within a mean follow-up period of 14.3 ± 5.1 months. The rate of sinus rhythm maintenance was 92%. PV reconnection was observed in 2 patients. None of the patients had postoperative atrial flutter.

**Conclusions:**

Selecting patients for cryoablation according to contrast-enhanced CT findings made the procedure easier to perform, leading to improved early success rates and clinical course.

## Introduction

Ablation of atrial fibrillation (AF) is mainly performed using pulmonary vein isolation (PVI). PVI is commonly performed with radiofrequency (RF), but PVI with cryoballoon has been reported to be an easier and shorter procedure without reconnection and lower risk of re-ablation [1, 2].

Recent findings on the outcomes of cryoablation of persistent AF [3] suggest that cryoablation may play an important role in the treatment of persistent AF. However, insufficient cryoablation due to anatomical factors or the location in the pulmonary vein (PV) limits the usefulness of PVI with cryoablation [4]. Inadequate isolation of the pulmonary vein carina during ablation leads to AF recurrence [5].

The purpose of this study was to determine whether patient selection according to contrast-enhanced CT improves the early success rate and prevents the recurrence of AF in PVI with cryoablation.

## Methods

### Study design and patients

**Fig. 1:**
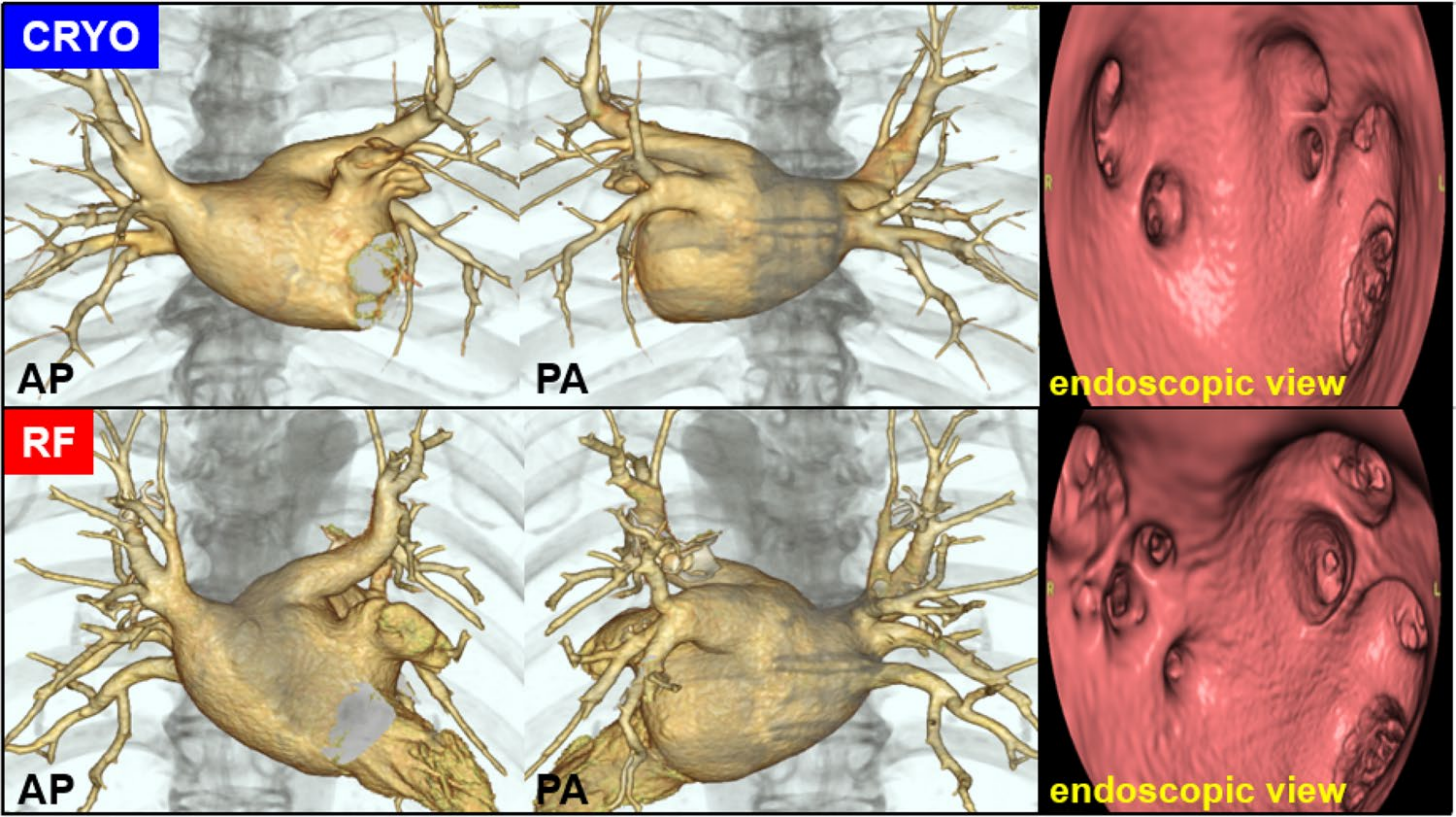
Anatomical morphology of PV and selection of treatment device. The figure shows the selection criteria for the treatment device used in this study. Among the patients with PVs that could be isolated with a 28-mm cryoballoon, those with a common trunk or the three branches of the pulmonary vein are excluded. For adequate isolation of the carina, patients with an overextended carina are also excluded

This prospective, single-center observational study evaluated patients who underwent ablation for paroxysmal atrial fibrillation in our hospital between July 2019 and November 2020. Patients contraindicated for using contrast medium due to renal dysfunction and allergy were excluded. A treatment plan was established for each remaining patient based on the visual inspection of the results of preoperative contrast-enhanced CT by two independent electro physiologists. Inclusion criteria were paroxysmal AF with simple anatomy that would optimize balloon fit in the PV. The anatomical morphology of the PVs in the selected patients is shown in Fig. 1. The cryoablation plan was developed according to the size, branches, and shape of the PV. The eligibility for cryoablation was determined based on the following criteria: (i) the PV can be completely occluded with a 28-mm balloon; (ii) cryoablation of the superior and inferior PVs involves adequate isolation of the carina; and (iii) the absence of the common trunk or the three branches of the PV.

### Ablation protocol

Antiarrhythmic drugs were discontinued at five half-lives before ablation. None of the patients had been receiving amiodarone. Ablation was performed under sedation with propofol and dexmedetomidine hydrochloride. During the ablation procedure, heparin was administered to achieve an activated clotting time (ACT) of ≥ 300 s. A 12.5F delivery sheath (FlexCath Advance; Medtronic, Inc., Minneapolis, MN) was introduced into the left atrium (LA) over-the-wire along with the cryoballoon catheter (Arctic Front Advance, Medtronic). A dedicated inner-lumen mapping catheter (Achieve, Medtronic) was commonly used to deliver the cryoballoon and sheath. Three-dimensional (3D) mapping was performed using an electroanatomic 3D mapping system (Ensite Velocity, Abbott Laboratories, Abbott Park, IL, USA). A TactiCath SE irrigation catheter (Abbott Laboratories, Abbott Park, IL, USA) was used for additional RF ablation.

All patients initially underwent PVI with a cryoballoon. Successful PVI was defined as the presence of bidirectional block of the PVs. To prevent phrenic nerve palsy, diaphragm muscle action potentials were monitored using compound motor action potential (CMAP) during cryoablation of the right PV. Continuous phrenic nerve stimulation of the superior vena cava (SVC) was performed. The CMAP of the diaphragm was recorded while monitoring the diaphragm twitching. To prevent phrenic nerve palsy, cryoablation was stopped when the amplitude of the CMAP decreased.

Next, isoproterenol (ISP) was administered after PVI to evaluate the complete acute PVI lesion and induce non-PV foci. The patients received intravenous ISP (6 μg/min for 5 min). Non-PV foci were defined as those that induced AF, supraventricular tachycardia, and frequent or repetitive premature atrial contractions. In the case of induction of non-PV foci, the origin was mapped using a 20-pole ring electrode catheter, and additional ablation was performed. In addition to the induction by a drug, induction of AF by rapid atrial pacing and spontaneous AF after defibrillation were also evaluated. The evaluation and the procedure were completed within approximately 15 min (i.e., duration of efficacy) after the administration of ISP.

In addition to PV loss of potential, pacing in the PV on both sides (bilateral) and the carina areas were performed in all cases; we ensured no LA capture, and the exit block was complete. Finally, blocks in both directions were checked.

### Patient follow-up

The patients were followed up using self-reported outpatient symptoms and 12-lead electrocardiogram (ECG) at 1, 3, 6, and 12 months after ablation. Holter ECG monitoring was also performed. Holters were certainly performed during the follow-up period, but the frequency was determined by the attending physicians. The average time for Holters since ablation was 56.6 ± 28.2 days. AF recurrence was defined as an ECG recording of AF of more than 30 s, with or without drug therapy. AF and other atrial tachycardias during the blanking period (i.e., during the first 3 months following treatment) were excluded from the recurrence analysis.

### Statistical analyses

Continuous data were expressed as the mean ± standard deviation for the normally distributed variables and as the median (25th and 75th percentiles) for the non-normally distributed variables. Kaplan–Meier curves were used to visualize the patterns of outcome occurrence, defined as recurrence. All statistical analyses were performed using SPSS 22.0, for Windows (IBM Japan, Ltd., Tokyo, Japan).

## Results

### Patient characteristics

Among 247 people with paroxysmal AF, a total of 50 patients including 33 male patients (66%) with a mean age of 68.4 ± 10.2 years were enrolled. The patient characteristics are shown in Table 1. The mean duration of AF was 8.9 ± 15.6 months. The mean left ventricular ejection fraction was 65.2 ± 7.1%, and the mean left atrial diameter was 35.4 ± 7.1 mm. The mean BNP level was slightly high (73.5 ± 101.4 pg/ml).

**Table 1:** Patient characteristics (*n* = 50)

Parameters	
Sex (male/female)	50 (33/17)
Age (years)	68.4 ± 10.2
Duration of AF (months)	8.9 ± 15.6
Hypertension, *n* (%)	30 (60%)
Diabetes, *n* (%)	16 (32%)
Structural heart disease, *n* (%)	6 (12%)
CHADS2	1.3 ± 1.0
CHA2DS2-VASc	2.5 ± 1.4
LVEF (%)	65.2 ± 7.1
LAD (mm)	35.4 ± 7.1
Ccr (mL/min)	65.5 ± 22.2
BNP (pg/mL)	73.5 ± 101.4

^*a*^*AF*, atrial fibrillation; *LVEF*, left ventricular ejection fraction; *LAD*, left atrial diameter; *BNP*, brain natriuretic peptide

### Ablation procedure

Details of the ablation procedure are shown in Table 2. The mean duration of the procedure was 78.8 ± 15.5 min, and the mean fluoroscopy time was 18.0 ± 5.7 min. Among the 200 PVS (4 PVs per patient), 192 PVs (96%) were isolated with a single cryoablation. Of the eight PVs with incomplete isolation with cryoablation, six were successfully isolated with additional cryoablation immediately after. Two PVs with incomplete isolation with cryoablation (2 patients, 4%) were subjected to additional PVI with RF ablation in the same session. Both patients underwent PVI with a single ablation at the bottom of the right inferior PV (RIPV bottom). Dormant conduction after ISP administration was observed in 5 patients (10%). The sites of reconnection were the RIPV bottom in 4 patients and the anterior aspect of the left superior PV (LSPV anterior) in 1 patient. Two patients underwent successful PVI with cryoablation, whereas three patients required re-isolation with additional RF ablation. Overall, 8 patients (16%) underwent additional non-PV ablation with ISP infusion. Three patients (6%) developed common AFL and cavotricuspid isthmus (CTI) was ablated. Meanwhile, 1 patient (2%) had atrioventricular nodal re-entrant tachycardia (AVNRT) and therefore underwent additional slow pathway ablation. There were 3 patients (6%) who underwent SVC isolation, whereas 1 patient (2%) underwent focal ablation of the coronary sinus.

**Table 2:** Ablation parameters

Parameters	
Procedure time (min)	78.8 ± 15.5
Fluoroscopy time (min)	18.0 ± 5.7
Isolation in one freezing, *n* (%)	192/200 (96%)
Isolation in second freezing, *n* (%)	198/200 (99%)
+ RF ablation for PVI, *n* (%)	2/50 (4%)
Dormant conduction, *n* (%)	5 (10%)
Isolation by cryoablation, *n* (%)	2 (4%)
Isolation by RF ablation, *n* (%)	3 (6%)
Non-PV foci, *n* (%)	8 (16%)
+ CTI block line, *n* (%)	3 (6%)
+ SVC isolation, *n* (%)	3 (6%)
+ Slow-pathway ablation, *n* (%)	1 (2%)
+ Focal ablation, *n* (%)	1 (2%)

^*a*^*RF*, radiofrequency; *PV*, pulmonary vein; *PVI*, pulmonary vein isolation; *CTI*, cavo-tricuspid isthmus; *SVC*, superior vena cava

### Confirmed of ablation area

**Fig. 2:**
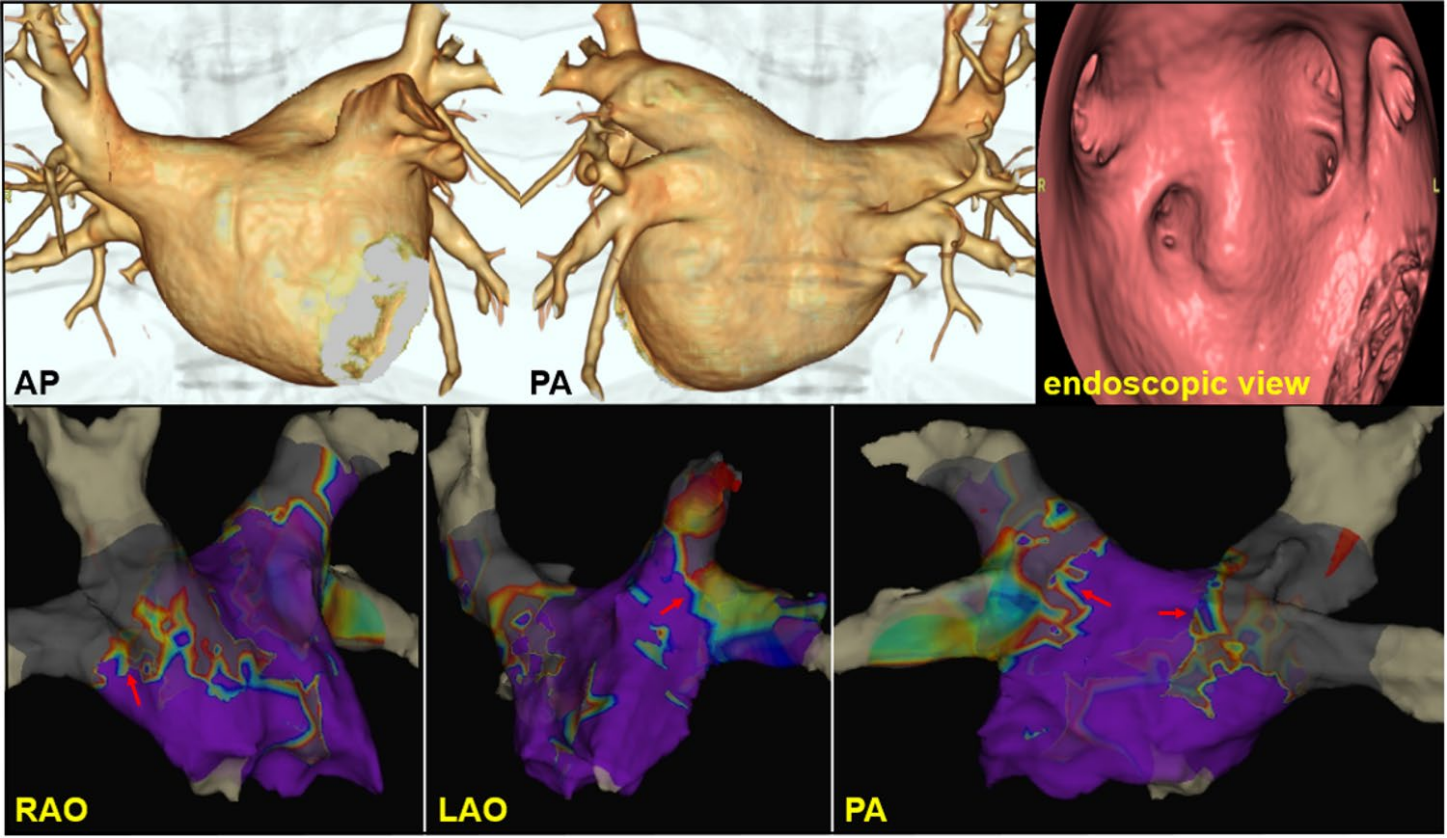
Ablation area after cryoablation. An 81-year-old man with a left atrial diameter of 41 mm underwent successful PVI with a single cryoablation for each of the four PVs. A voltage map (range, 0.1–0.2 mV) is created posttreatment to confirm the ablation area. The isolation range with adequate isolation of the carina is equivalent to that in RF ablation. The red arrow indicates the isolation line in the carina area

Seven patients (14%) were created postoperative voltage map to confirm the ablation area. We present one of those cases. The patient was an 81-year-old man with a left atrial diameter of 41 mm. He underwent successful PVI with a single cryoablation for each of the four PVs. After the treatment, a voltage map (range, 0.1–0.2 mV) was created to confirm the ablation area (Fig. 2). The voltage map showed wide antral PVI with adequate isolation of the carina. In all patients, pacing was confirmed in the bilateral PVs and carina, whereas pacing from the PV carina did not capture the LA.

### Long-term follow-up

**Fig. 3:**
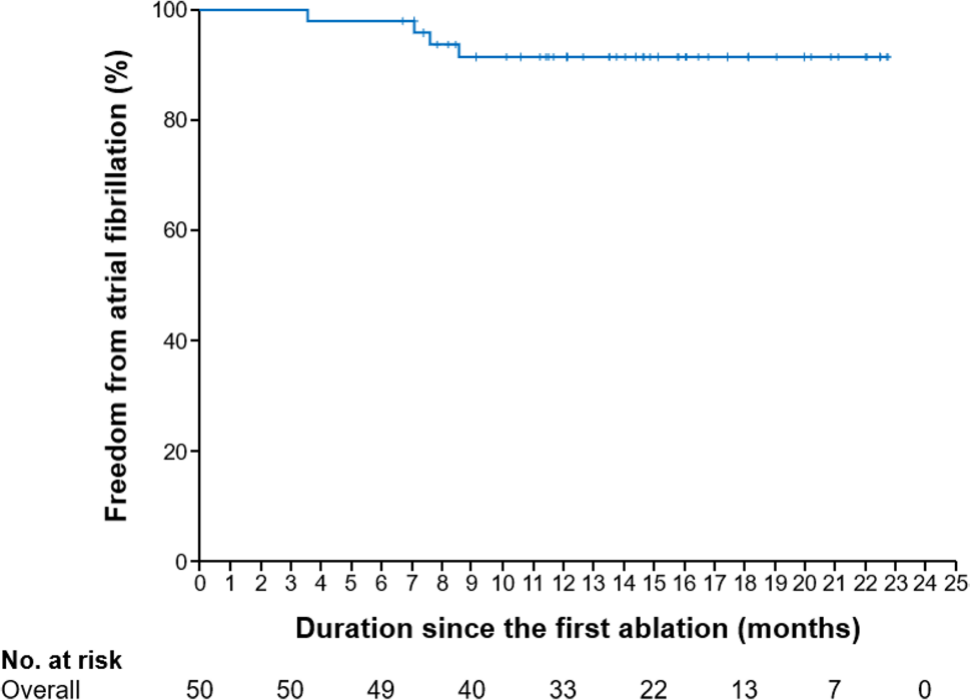
Resolution of atrial fibrillation. The rates of sinus rhythm maintenance after cryoablation. Four patients had AF recurrence during the mean follow-up period of 14.3 ± 5.1 months. The rate of sinus rhythm maintenance is 92%

The clinical course of AF resolution after cryoablation is shown in Fig. 3. Within the mean follow-up period of 14.3 ± 5.1 months, 4 patients had AF recurrence. The rate of sinus rhythm maintenance was 92%. Of these patients, three patients underwent second ablation, and two experienced PV reconnection. Of the two patients who had reconnection of the left superior PV, one patient had reconnection of the left inferior PV. The other patient had no PV reconnection. In all patients, the non-PV foci were not induced by the first or second ablation. The first cryoablation was PVI alone. The patients underwent a re-isolation with RF ablation. The isolation range of RPV was extended to patients without PV reconnection. No recurrence was observed thereafter. None of the patients experienced recurrence of AFL. Therefore, only three patients with AF underwent second ablation. Table 3 lists the adverse events after ablation. Except for one case of postoperative hematoma at the access site and one case of pneumothorax, no other major adverse events were observed.

**Table 3:** Adverse events

Parameters	
Hematoma at access site	1 (2%)
Arteriovenous fistula or aneurysm at access site	0 (0%)
Pneumothorax	1 (2%)
Phrenic nerve damage	0 (0%)
Pericarditis	0 (0%)
Fluid overload	0 (0%)
Sedation-related complication	0 (0%)
Cardiac tamponade	0 (0%)
Transient ischemic attack or stroke	0 (0%)
Atrioesophageal fistula	0 (0%)
Pulmonary vein stenosis	0 (0%)

^*^Data are presented as *n* (%)

## Discussion

### Main findings

The use of contrast-enhanced CT as a guide for cryoablation in this study led to a successful procedure and good long-term outcomes compared to past reports [6, 7]. The advantages of this method are as follows: (1) Although the treatment indication is determined based on contrast-enhanced CT images, the method requires convenient visual inspection but not measurement. (2) The patients were included based on the selection criteria for cryoablation considering the shape of the PV, and thus a high rate of successful PVI with cryoablation alone was achieved. (3) The procedure also allowed the isolation of the carina. (4) Although the procedure also involves the elimination of dormant conduction and induction of non-PV foci with ISP infusion, the overall duration of the procedure is relatively short due to a lower time required for PVI. To our best knowledge, this is the first study of cryoablation in which the treatment strategy for AF ablation was determined based on the findings of contrast-enhanced CT.

### Usefulness of contrast-enhanced CT as a guide for PVI with cryoablation

Cryoablation has been reported to have similar efficacy and safety to radiofrequency ablation [1, 6]. The risk of re-ablation is also lower in cryoablation than in RF ablation [2]. Further, cryoablation has a lower incidence of PV reconnection, especially the superior PV [8]. However, cryoablation does not involve the simultaneous isolation of the superior and inferior PVs, and the carina region plays an important role in AF [9]. AF ablation without carina isolation is associated with a higher risk of AF recurrence [5]. In addition, a study that examined the isolation range after cryoablation using a voltage map showed better long-term outcomes in the group with adequate isolation of the carina [10]. Furthermore, insufficient cryoablation due to anatomical factors and the site of PV lowers the effectiveness of PVI [3]. Therefore, optimal patient selection is important for PVI with cryoablation.

To maximize the efficacy of cryoablation and achieve long-term PVI with adequate isolation of the carina, we used contrast-enhanced CT guidance in PV patients eligible for cryoablation. A high success rate of PVI with cryoballoon alone was achieved. The two patients with incomplete isolation by cryoballoon had PV connection at the bottom of the RIPV. Confirmation by pacing showed isolation of the carina in all the patients.

ISP is useful in identifying dormant conduction (DC) [11], which was also observed in this study in which ISP was used to increase the long-term effectiveness of PVI. Patients with DC in this study underwent re-isolation with a cryoballoon, focused on the site of conduction. This led to successful re-isolation with cryoablation alone in 2 of 5 patients (40%). Regarding the site of DC, 4 of the 5 patients (80%) that showed DC after PVI had RIPV bottom ablation. The PVI was also for the RIPV bottom in patients who required RF ablation, in line with a report of a high incidence of reconnection in RIPV bottom [12]. Additional ablation of the site as a DC may result in higher benefits, no patient who underwent a second ablation showed reconnection of RIPV in this study.

The incidence of non-PV foci ranges from 10 to 20% [13–16]. Patients with non-PV foci have poor ablation outcomes [16, 17]. However, patients with successful ablation of non-PV foci have better outcomes than those with incomplete ablation of non-PV foci [17, 18]. Therefore, ablation should only be attempted in patients with paroxysmal or persistent AF originating from non-PV foci. In this study, 8 patients, including 3 patients with common AFL and 1 patient with AVNRT (16%), underwent additional ablation. Ablation of non-PV foci is a difficult procedure. However, since we performed PV isolation, swiftly isolating the PV and reliably performing cryoablation elucidated the role of cryoablation in this study. We considered that subsequent non-PV foci ablation would take time, and the possibility of additional ablation of non-PV foci may prolong prognosis.

No postoperative AFL recurrence was observed in the present study. This may be due to the following reasons: (i) there was no conduction gap due to adequate isolation of the carina, and (ii) patients in this study were without anatomical deformation of the PV, leading to a lower risk of leaving a conduction gap.

Concerning future applications of this treatment, there are a report that performing cryoablation with intracardiac echo (ICE) and Doppler to reduce exposure to radiation [19]. In cases selected for this protocol, it might be more effective to utilize ICE due to the suitable morphology of the PV as this allows the procedure to be performed simply.

### Study limitations

This study had some limitations. First, the number of patients was small and there was no control group because this was a prospective study conducted at a single institution. Due to the methods used in this study, treatment was initiated at a relatively short time after initiating cryoablation. Unfortunately, there was no control group. The study findings should be confirmed in further large-scale research. Second, the detailed isolation range was unknown because not all patients underwent confirmation of the ablation area using the voltage map. However, several patients were confirmed to have adequate isolation of the carina. An adequate carina isolation can be expected in most patients in this study because they were selected after confirmation of the shape of the PV. Confirmation of pacing from the PV carina showed carina isolation in all patients. Last, the follow-up by Holter ECG was performed at the discretion of the attending physician, which may have caused variations in the follow-up accuracy due to non-compliance with the protocol.

## Conclusions

Selecting patients for cryoablation according to contrast-enhanced CT findings optimizes the outcomes of PVI with cryoablation, allowing for easy isolation of the PV including the carina and ultimately leading to an improved success rate and clinical course.

## Data Availability

Not applicable.
